# An ITGA11 expressing subpopulation as predictor for the donor-specific osteogenic capacity of stromal cells

**DOI:** 10.1038/s41413-026-00536-2

**Published:** 2026-06-15

**Authors:** Ali Jasim Mohammad Jamil, Kaja Madsen, Souad Daamouch, Birgitte Villadsen, Chao Ma, Shakespeare Jeromdesella, Mikkel Ørnfeldt Nørgård, Emilie Grupe, Maria Bruun Jakobsson, Yuting Wang, Eri Takematsu, Liming Zhao, Stuart Barry Goodman, Lawrence Henry Goodnough, Charles Kwok Fai Chan, Thomas Levin Andersen, Moustapha Kassem, Alexander Rauch

**Affiliations:** 1 The Molecular Endocrinology & Stem Cell Research Unit (KMEB), Department of Endocrinology, Odense University Hospital, Odense, Denmark; 2 Department of Clinical Research, University of Southern Denmark, Odense, Denmark; 3 Department of Clinical Research, Molecular Bone Histology (MBH) lab and Danish Spatial Imaging Consortium (DanSIC), University of Southern Denmark, Odense, Denmark; 4 Department of Pathology, Odense University Hospital, Odense, Denmark; 5 Institute for Stem Cell Biology and Regenerative Medicine, Stanford University School of Medicine, Stanford, USA; 6 Department of Orthopedic Surgery, Stanford University School of Medicine, Stanford, USA; 7 Department of Surgery, Stanford University School of Medicine, Stanford, USA; 8 Department of Cellular and Molecular Medicine, University of Copenhagen, Copenhagen, Denmark; 9 College of Medicine and Health Sciences (CMHS)& Center for Biotechnology, Khalifa University, Abu Dhabi, UAE; 10 Steno Diabetes Center Odense, Odense University Hospital, Odense, Denmark

**Keywords:** Bone, Osteoporosis

## Abstract

Stromal progenitor cells of bone marrow origin are non-hematopoietic cells that give rise to osteoblasts and adipocytes in the postnatal organism. Marrow stromal cells (also known as mesenchymal stem cells - MSCs) are currently being employed in a large number of clinical trials for regenerative purposes post in vitro expansion. However, the clinical outcome has been variable, which might in part be due to the heterogeneity of the cells and the lack of a defined cell product with a molecular signature that favors tissue regeneration. In this study, we determined the cellular heterogeneity of primary stromal cultures and examined how inter-donor variation in subpopulation composition contributes to the differentiation potential of primary cultures. We profiled 136 014 stromal progenitors from 26 donors and identified 5 subpopulations that were linked to distinct bone-related pathways and genetic traits of bone mineral density and morphology. Abundance of one cluster characterized by high expression of *ITGA11* (integrin alpha-11) and genes related to matrix function, collagen organization, and elevated expression upon lineage commitment was positively correlated with osteoblastic differentiation capacity in vitro. In addition, ITGA11 protein expression in progenitor cells was a predictive marker for matrix mineralization in vitro and ectopic bone formation in vivo. Sorting stromal progenitors into ITGA11^high^ and ITGA11^low^ cells established cultures with high and low osteoblastic differentiation potential and revealed transcriptional differences reflective of the subpopulation-specific signature, which was not affected by siRNA-mediated knockdown of *ITGA11* expression. Our findings corroborate the presence of an extensive donor-dependent cellular heterogeneity that persists in cultured stromal cells, and that ITGA11 can be employed as a marker for isolating cells with high bone-forming potential, a feature likely to benefit clinical trials of bone regeneration.

## Introduction

Stromal cells of the bone marrow, also known as mesenchymal stem cells (MSCs), are non-hematopoietic cells with stemness features that contain progenitors with the ability to differentiate into various cell types, such as bone-forming osteoblasts, cartilage-forming chondrocytes, and fat-storing adipocytes.^1^ Stromal cells exhibit fibroblast-like morphology when cultured and expanded in vitro, and they have been implicated in several biological processes such as bone remodeling, regeneration, hematopoietic stem cell support, and modulating immune responses.^2,3^ Due to their ease of collection, ease of in vitro propagation, as well as immune modulatory properties, bone marrow stromal cells have been propelled to the forefront of regenerative medicine, where they are being explored as treatment options to improve regeneration in conditions like bone fractures and cartilage damage.^4,5^ However, the effectiveness of using “MSCs” for bone regeneration has been reported with inconsistent clinical outcomes, which may have been caused by the heterogeneity of cellular products used.^5,6^

Lineage tracing studies in mice have identified distinct cell populations expressing osterix,^7^ nestin,^8^ PTH related peptide,^9^ gremlin 1^10^ or the leptin receptor,^11^ that are capable of becoming osteoblasts. In addition to anatomical differences, these cells show unique features and context-dependent differentiation potential during bone development, growth, maintenance, and regeneration. Huge effort has been employed by multiple research groups in order to delineate the hierarchy of the osteogenic lineage in humans and to describe the differentiation potential of distinct stromal subpopulations.^12–14^ Using a defined panel of cell surface markers (CD45^−^, CD235^−^, CD31^−^, TIE2^−^, CD146^−^, PDPN^+^, CD164^+^, and CD73^+^ in humans), the laboratories of Longaker and Chan were able to define a SSC, in humans^13^ and mice,^15^ for which differentiation potential is limited to the osteochondral lineage. Recent extensive single-cell RNA-seq studies in developing human bone structures revealed a dedicated regional complexity of those human SSCs^16^ as well as osteochondral progenitors.^17^ Since stromal progenitor frequency is low,^18^ enrichment strategies have been employed to determine molecular signatures of human stromal progenitors with emphasis on lineage relation, distinct spatial localization, and differentiation potential.^19,20^ These data often coincide with anatomical distinct lineage trees, i.e., endosteal progenitors with higher levels of osteogenic genes and marrow progenitors with adipogenic gene expression.^19,20^ So far, knowledge of whether stromal cells from similar locations are heterogeneous and whether modulation of molecular profiles and composition between donors translates to altered cellular and physiological properties is limited.

Our group has performed a comprehensive characterization of primary bone marrow-derived stromal cells that exhibited a large inter-donor variation in terms of their osteoblast and adipocyte differentiation potential in vitro. These differences were in part related to clinical features such as lifestyle factors and sex, but also to morphological features of the cells, such as shape and geometry.^21,22^ Taking into consideration that stromal cells are heterogeneous in terms of function, skeletal sites, and differentiation hierarchy, it is questionable whether subject-specific cellular composition of primary cultures contributes to functional differences observed among donor-derived primary cultures.

In the current study, we explored in a more detailed fashion the intrinsic variation of stromal cell cultures employing scRNA-seq profiling in primary stromal cells from 26 donors. Here, we identified 5 cell populations with distinct molecular features, related to genetic and functional aspects of bone biology, and for which donor-specific composition was linked to differences in the osteoblastic differentiation potential. In particular, the abundance of an integrin alpha-11 (*ITGA11)*-expressing subpopulation was associated with high osteogenic potential in vitro and in vivo. While ITGA11-based sorting enriched for cells displaying this osteogenic gene signature, loss of ITGA11 did not perturb the underlying program, highlighting that ITGA11 displays but does not drive this transcriptional state.

## Results

### Study design and differentiation potential towards osteoblast and adipocyte lineage

**Fig. 1:**
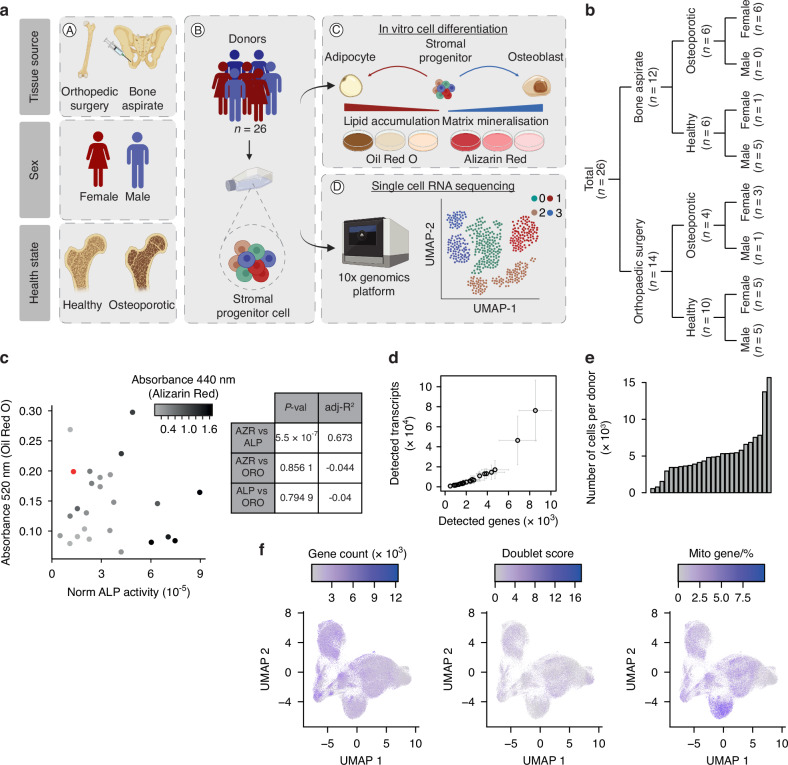
Cohort overview and donor-specific differentiation potential. **a** This study comprises 26 primary bone marrow-derived stromal cell cultures obtained at Odense University Hospital (OUH) from orthopedic surgeries or bone marrow aspirates Ⓐ and Ⓑ. Cultures of undifferentiated cells have been subjected to in vitro differentiation towards osteoblasts and adipocytes Ⓒ and single cell RNA sequencing Ⓓ. Generated in Biorender. **b** Tree showing source of biopsies, osteoporosis status, and sex of the donors. **c** 26 primary stromal cell culture from **a** were subjected to osteogenic differentiation followed by measuring alkaline phosphatase (ALP) activity at day 7 and matrix mineralization (Alizarin Red - AZR) at day 14 or to adipogenic differentiation and lipid droplet formation (Oil Red O - ORO) was quantified at day 14. Scatter plot quantifying lipid droplet formation over normalized ALP activity, with color code presenting matrix mineralization. Table shows statistics from linear regression models. **d** scRNA-seq quality measures showing mean number of transcripts over genes for each donor. Arrow bars represent standard deviation. **e** Bar plot showing number of scRNA-seq quality passing cells for each donor. **f** UMAP plot quantifying number of detected genes, doublet score, and percentage of mitochondrial genes for the 136 014 cells in the scRNA-seq data set

Stromal cells of bone marrow origin can be obtained from various skeletal sites, and their subsequent differentiation potential is influenced by multiple donor attributes such as sex, lifestyle factors, and disease state,^22^ and can in part be predicted by morphological features of cells and nuclei.^21^ To explore a common molecular signature predictive for osteogenic potential of primary human cells, we combined molecular profiling by single-cell RNA-seq with in vitro differentiation assays from primary cultures derived from women and men, with and without osteoporosis, that were established from bone biopsies removed during orthopedical surgery or from marrow aspirates of the iliac crest (Fig. 1a, b). Determining the osteogenic and adipogenic potential in vitro (Fig. 1c), we could show large inter-donor variations in the ability of the cultures to differentiate into osteoblasts as evaluated by ALP activity or matrix mineralization, as well as to adipocytes as estimated by lipid droplet formation. Within our cohort, there was no relation between adipogenic potential and osteogenic capacity of the cells.

Using the 10x Genomics platform, we performed scRNA-seq on those 26 primary stromal cell cultures prior to differentiation. Since libraries had different efficiencies in transcript (Fig. 1d) and cell capturing (Fig. 1e), we performed rigorous quality control (Fig. 1f) to finally integrate 136 014 high-quality cells.

### Stromal cell subpopulations have distinct associations to bone biology

**Fig. 2:**
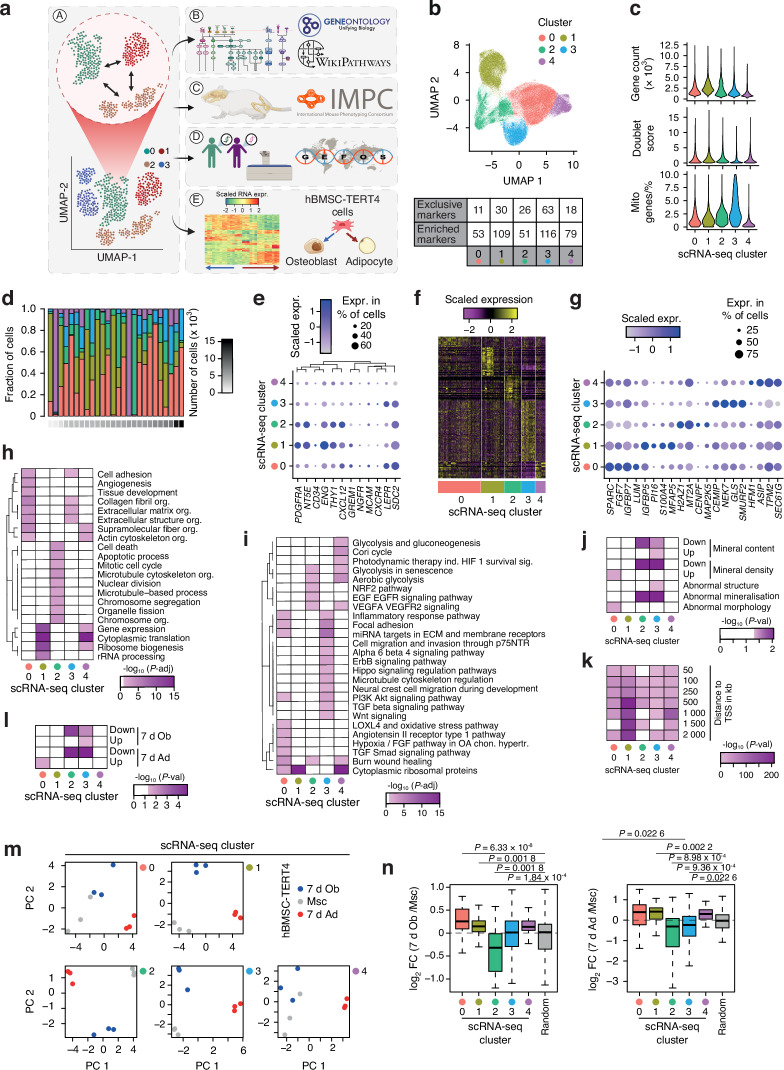
Heterogeneity among primary human stromal cells is linked to bone function. **a** Schematic representation of heterogeneity between subpopulations Ⓐ. Cluster-specific genes were subjected to enrichment analysis using pathways repositories Ⓑ, X-ray-based phenotyping data of knockout mouse lines Ⓒ, genomic distribution of bone mineral density associated-variants Ⓓ, and gene expression data from hBMSC-TERT4 cells during osteogenic and adipogenic differentiation Ⓔ. Generated in Biorender. **b** UMAP plot of 136 014 cells from 26 primary cultures in Fig. 1. Table shows number of genes with enriched or exclusive expression. **c** Violin plots quantifying number of detected genes, doublet score, and percentage of mitochondrial genes. **d** Stacked bar plot for the distribution of cells. **e** Dot plot highlighting cluster-specific expression of classical MSC markers and genes tracing osteoblast progenitors in mice. **f** Heat-map showing scaled expression levels of exclusive cluster markers. **g** Dot plot highlighting cluster-specific gene expression patterns. **h** Gene ontology analysis for enriched cluster markers (cutoff Benjamini–Hochberg *P*-adj < 0.01). **i** rWiki-pathway analysis for enriched cluster markers (cutoff Benjamini–Hochberg *P*-adj < 0.01). **j** Overlap between enriched cluster markers and genes linked to bone mineral content or density as well as abnormal bone structure, mineralization, or morphology by the International Mouse Phenotyping Consortium (hypergeometric test cutoff *P*< 0.05). **k** Heat map showing the distance dependent and chi-square-based enrichment of eBMD-associated SNPs^26^ near enriched cluster markers (cutoff *P* < 10^-5^). **l** Overlap between enriched cluster markers and genes that show differential expression in hBMSC-TERT4 cells after 7 days of osteogenic or adipogenic differentiation^27^ (hypergeometric test cutoff *P* < 0.05). **m** PCA-plots based on enriched cluster markers using gene expression data of hBMSC-TERT4 cells prior to and after 7 days of osteogenic and adipogenic differentiation.^27^ **n** Box plot quantifying changes in gene expression after 7 days of osteogenic (left) or adipogenic (right) differentiation in hBMSC-TERT4 cells for enriched cluster markers. Random gene set (*n* = 100) was used for unpaired two-tailed Wilcoxon–Mann–Whitney test

Using the 136 014 cells from the 26 donors we first explored to degree of cellular heterogeneity among cultured stromal cells and questioned whether genes specific to stromal cell subpopulations were linked to distinct pathways, genetic traits of bone biology, and gene networks implicated in cellular differentiation along the osteogenic or adipogenic lineage (Fig. 2a). We identified 5 subpopulations among cultured stromal cells (Fig. 2b) that were neither related to quality metrices (Fig. 2c) nor number of recovered cells in the individual libraries (Fig. 2d). Importantly, we were able to recover cells from multiple subpopulations for each donor (Fig. 2d), highlighting the validity of stromal cell heterogeneity in vitro. Focusing on marker genes suggested by the international society for cellular therapy (*THY1* encoding CD90, *NT5E* encoding CD73, *ENG* encoding CD105^23^), from lineage tracing studies in mice (*LEPR*,^11^ *CXCL12*,^24^ and *GREM1*^10^) and from our previous study using cell surface marker expression to predict osteogenic capacity of stromal cells (*PDGFRA*, *CD34*, *NGFR* encoding CD271, *MCAM* encoding CD146, *CXCR4*, and *SDC2* encoding CD362^22^), we observed a high variability in marker gene expression across all cell clusters (Fig. 2e). Performing differential gene expression analysis, we identified a panel of exclusive, highly expressed in cluster of interest compared to all other clusters individually, as well as enriched markers, highly expressed in cluster of interest against all other cells combined (Fig. 2b table). With focus on exclusive markers (Fig. 2f, g), we observed that marker gene expression was quantitatively rather than qualitatively different across clusters.

To explore functional differences between clusters, we focused on enriched markers (Fig. 2b table) and first performed enrichment analysis with pathway repositories. Using gene ontology (Fig. 2h) and Wiki pathways (Fig. 2i), we annotated the clusters to be implicated in various biological functions. Cluster 0 and 3 represent cells linked to collagen production, extracellular matrix organization, cytoskeleton organization, and gene signatures of PI3K signaling. While cluster 0 was specific to angiogenesis and hypoxia signaling, cluster 3 showed signatures of various signaling pathways such as WNT, Hippo, and TGF-β. Cluster 2 cells were enriched in genes associated with proliferation and senescence in line with a gene signature of elevated NRF2 activity. Cluster 4 genes were involved in actin cytoskeleton organization, as well as anabolic and catabolic glucose metabolism. Despite cluster 1 showing a substantial number of cluster-specific genes, associations were limited to translation and rRNA processing. Based on these analyzes, we postulated that cells from cluster 0 and 3 to exhibit an osteoblastic phenotype while cells of the other clusters might be linked to proliferation and metabolic phenotypes.

To test whether cluster 0 and cluster 3 genes are preferentially linked to bone function, we associated cluster-specific gene signatures with genetic data using phenotypes of thousands of knockout mouse models and GWAS for human bone mineral density. Grouping genes according to X-ray-based phenotypes from IMPC, a resource from large-scale phenotyping of knockout mice,^25^ we found distinct enrichments for cluster 0, 2, and 3 (Fig. 2j), indicating bone-selective properties of the identified cell populations. Using GWAS summary statistics from estimated bone mineral density,^26^ we found a strong enrichment of bone mass-associated sequence variations in the vicinity of cluster-specific genes (Fig. 2k), highlighting that all subpopulations might be equally important for bone mass homeostasis in humans.

Finally, we questioned whether those cluster-specific genes are indicative of cellular commitment using lineage-specific gene signatures of differentiating human stromal cells.^27^ Similar to knockout-associated phenotypes, we found genes specific for cluster 0, 2, and 3 to be dynamically expressed during osteoblast or adipocyte differentiation of hBMSC-TERT4 cells^28^ (Fig. 2l), and again with distinct patterns. Importantly, all cluster-specific gene signatures were able to demarcate osteogenic, adipogenic, or undifferentiated hBMSC-TERT4 cells (Fig. 2m), implying that genes defining stromal subpopulations are subject to lineage-selective transcriptional remodeling. To determine lineage-selectivity of cluster-specific gene signatures, we quantified expression dynamics upon osteogenic (Fig. 2n left panel) or adipogenic (Fig. 2n right panel) commitment of hBMSC-TERT4-cells. Here, cluster 0 genes were most strongly induced, and cluster 2 genes were most strongly repressed during both osteoblast and adipocyte differentiation. Thus, cluster-specific gene signatures seem to be generally linked to cellular commitment and not subject to lineage-specific regulation. Taken together, primary stromal cells are heterogeneous in culture with molecularly defined subpopulations that can be identified in various donors, and that have implications in lineage commitment as well as bone homeostasis-related pathways and skeletal genetic traits.

### Donor specific subpopulation abundance aligns with differentiation properties

**Fig. 3:**
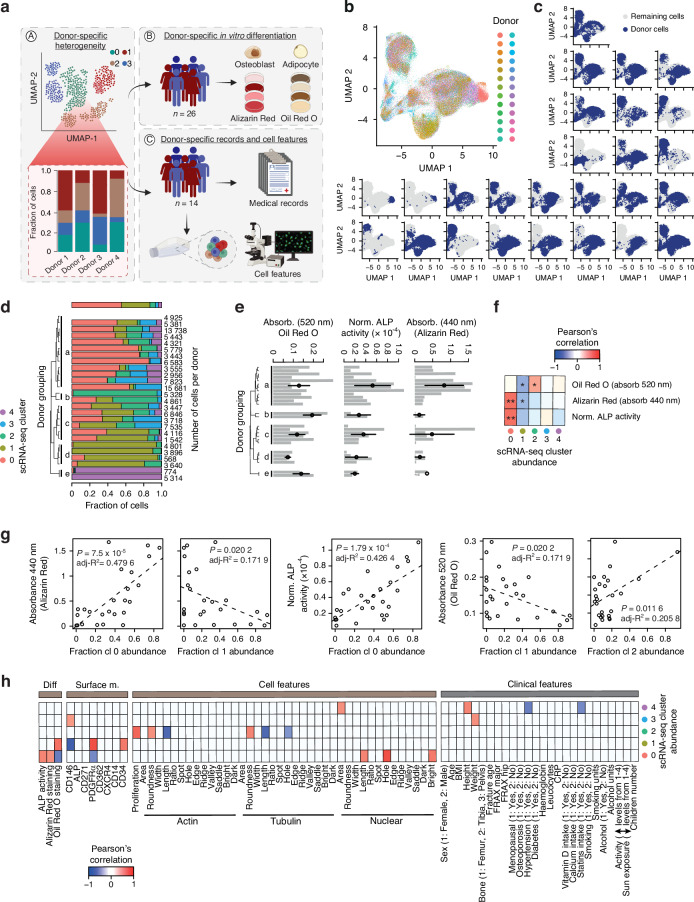
Donor-specific abundance of stromal cell subpopulations is linked to osteogenic differentiation. **a** Schematic representation of inter-donor heterogeneity in subpopulation composition Ⓐ. For each donor we correlated the relative abundance of stromal cell subpopulations with the osteogenic and adipogenic differentiation potential Ⓑ and with previously obtained clinical^22^ and cellular^21^ features of the donors Ⓒ. Generated in Biorender. **b** UMAP plot highlighting each individual donor. **c** 26 UMAP plots highlighting the cells of a single donor in blue, while the remaining cells in the data set are shown in light grey. **d** Stacked bar plots quantifying the distribution of cells across the 5 clusters for each of the 26 donors. Dendrogram shows hierarchical clustering of the relative cluster abundancies. Cells per sample is indicated. **e** Bar plot quantifying matrix mineralization (Alizarin Red), lipid droplet formation (Oil Red O), and ALP activity of the 26 primary samples grouped based on hierarchical clustering of relative cluster abundancies in Fig. 2d. **f** Heat map showing the Pearson’s correlation between donor-specific relative cluster abundance and differentiation outcomes. ^*^P < 0.05, ^**^P < 0.01 using linear regression model. **g** Scatter plot showing matrix mineralization (Alizarin Red) and ALP activity over relative cell abundance in cluster 0, as well as lipid droplet formation (Oil Red O) over cell abundance in cluster 1 and 2. Statistics from linear regression. **h** Of the 26 samples, 14 were studied previously in terms of cell morphology^21^ as well as cell surface molecule expression and clinical records.^22^ Heat map showing Pearson’s correlation for significant associations (linear regression with *P* < 0.05) between the listed parameters and relative cluster abundance

Having established that primary stromal cultures are heterogeneous, we questioned whether subpopulation abundance is linked to differentiation potential as well as previously determined clinical^22^ and cellular^21^ features in a donor-specific manner (Fig. 3a). Focusing on the individual donors (Fig. 3b, c), there was a considerable inter-donor variation regarding subpopulation abundance (Fig. 3d). Using subpopulation abundance for hierarchical clustering, we identified three groups largely dominated by a single cluster, i.e., cluster 1, 4, or 2. On the other hand, there were two larger groups in which donors had cells from multiple clusters in similar amounts. Interestingly, this grouping separated cultures with distinct osteogenic versus adipogenic potential (Fig. 3e). We therefore questioned whether the cellular abundance within a single cluster predicts donor-specific differentiation potential. Using Pearson’s correlation, we observed that ALP activity and mineralization capacity in vitro were associated with increasing abundance of cells in cluster 0, while enhanced adipogenic differentiation potential was associated with higher cell number in cluster 2 and lower cell number in cluster 1 (Fig. 3f, g). In contrast, abundance of cells in cluster 3 was not associated with the osteogenic capacity of the cells. We therefore could only affirm that cluster 0 cell abundance was linked with cellular differentiation.

Among the 26 primary cultures subjected to scRNA-seq in the current study, 14 had previously been examined for their differentiation potential as well as donor-specific cellular features^21^ and clinical characteristics.^22^ As expected, we observed that cells in cluster 2, expressing cell cycle-related genes, correlated with the proliferative capacity of the cultures (Fig. 3h). Importantly, percentage of cells in cluster 0 correlated positively with osteogenic and negatively with adipogenic differentiation potential and was associated with features of nuclear morphology such as nucleus length and texture (Fig. 3h), two features that had previously been shown to be predictive for osteogenic differentiation.^21^ In addition, more cells in cluster 1 were associated with a decline in adipogenic potential, a decrease in CD164-positive cells, and an increase in PDGFRα-positive cells, both previously linked to adipogenic differentiation.^22^ Cell numbers in cluster 3, were not associated with cellular differentiation but with the number of CD146-positive cells, a characteristic of osteoprogenitor cells.^22^ We had also reported that cell roundness based on actin staining as well as geometry and texture based on tubulin staining, are predictors of osteogenic and adipogenic differentiation.^21^ In the current study, they were linked to the abundance of cluster 2 cells, which aligns with their elevated expression of genes involved in cytoskeleton remodeling (Fig. 3h).

While scRNA-seq-based cellular abundance in cluster 0 and 1 correlated with osteogenic and adipogenic capacity determined in the current and previous studies,^21,22^ we did not observe any correlation with the clinical characteristics of the donors, which could in part be due to the limited sample size (*n* = 14). Likewise, previous reported clinical predictors of osteogenic and adipogenic differentiation, such as sex, osteoporosis, vitamin D supplementation, or alcohol consumption, were not associated with cell abundance for any of the clusters.

Taken together, primary cultures of human stromal cells showed a high degree of inter-donor-specific cellular heterogeneity, and the abundance of a subpopulation enriched in genes associated with extracellular matrix remodeling is predictive for cellular osteogenic potential. In addition, the molecular profiles defined in the current study are associated with cellular features such as PDGFRα or CD146 surface marker expression as well as nuclear morphology but not with clinical determinants such as sex or osteoporosis.

### ITGA11 and CD73 as stromal cell surface markers with high and low osteogenic potential

**Fig. 4:**
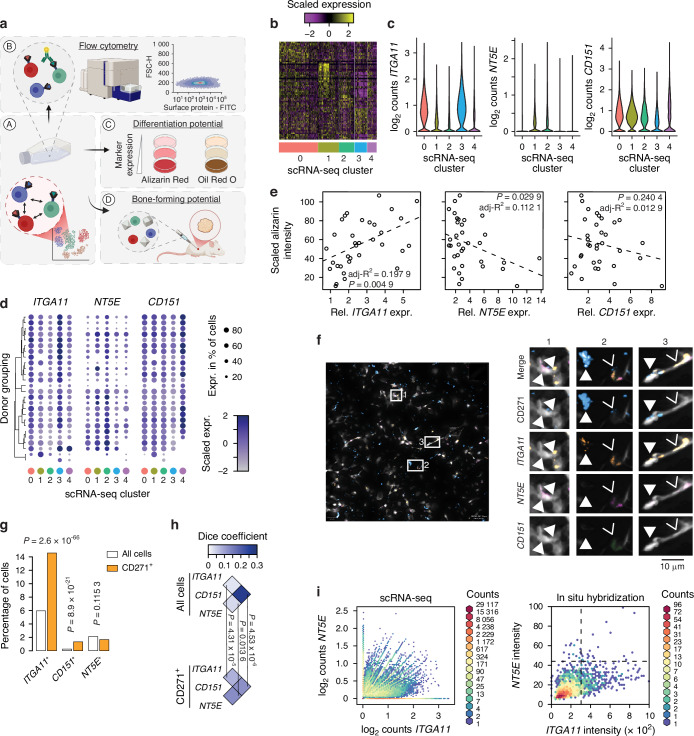
*ITGA11* expression marks stromal cells of cluster 0. **a** Scheme illustrating the identification of surface molecules specific for the scRNA-seq clusters Ⓐ. Primary cultures were quantified for the expression of surface markers using flow cytometry Ⓑ, in vitro differentiation potential Ⓒ, and bone forming capacity in a xenograft model Ⓓ. Generated in Biorender. **b** Heat map showing cluster-specific (enriched markers in Fig. 2b) expression patterns of putative cell surface molecules.^29^ **c** Violin plot showing cluster-specific expression patterns of *CD151*, *NT5E* (encoding CD73), and *ITGA11*. **d** Dot plot showing cluster specific expression of *CD151*, *NT5E* (encoding CD73), and *ITGA11* for each individual donor. Row clustering was taken from Fig. 2d. **e** Scatter plot showing the *CD151*-, *NT5E*- and *ITGA11*-mRNA expression levels of 34 primary cultures and their previously obtained osteogenic capacity in terms of matrix mineralization.^21^ Target gene expression was normalized to *GAPDH*. Statistics from linear regression. **f** Multiplex in situ hybridization of *CD151* (FITC), *ITGA11* (TxR), and *NT5E* (Cy5) together with immune staining of CD271 (Cy3) on an iliac crest bone biopsy of a healthy individual. Inserts highlight cells with co-expression of *NT5E* and *ITGA11* (open arrow heads) and cells which express either *ITGA11* or *NT5E* (filled arrow heads). **g** Quantification of *ITGA11*, *CD151*, and *NT5E* (encoding CD73) expressing cells among all (*n* = 33 163) and CD271^+^ cells (*n* = 2 639) from Fig. 4f. *P*-values from proportional *z*-tests **h** Heat map quantifying the pairwise overlap of *ITGA11*, *CD151*, and *NT5E* (encoding CD73) expressing cells among all and CD271^+^ cells from staining in Fig. 4f using dice coefficient. *P*-values from proportional *z*-tests. **i** Density scatter plots comparing *ITGA11* and *NT5E* (encoding CD73) mRNA expression levels in scRNA-seq data of 26 primary cultures (left panel) and by in situ hybridization within CD271^+^ cells of the iliac crest bone biopsy in Fig. 4f

Having identified a cell subpopulation with characteristics of an osteoblastic signature, we examined for the presence of surface molecules to be able to prospectively characterize primary cultures for their osteogenic capacity in vitro and in vivo (Fig. 4a). Using a catalogue of putatively expressed surface proteins^29^ we identified cluster specific surface proteins (Fig. 4b). We next choose ITGA11, CD73 (encoded by *NT5E*), and CD151 due to their opposing and partially overlapping expression patterns, with *ITGA11* being highly expressed in cluster 0 and 3, *NT5E* in 2 and 1, and *CD151* in cluster 0, 1, 2, and 4 (Fig. 4c). Importantly, these cluster-specific patterns were not driven by individual donors but reproducible across almost all subjects, except for the three subjects which mainly had cells from cluster 2 or 4 (Fig. 4d). ITGA11 is a transmembrane integrin that binds to osteolectin and thereby promotes WNT-signaling.^30^ CD73 is a known MSC-marker^23^ and primarily hydrolyzes AMP to produce adenosine for the purinergic signaling pathway.^31^ Although poorly studied in stromal cells, CD151 is a tetraspanin that improves post-binding adherence strength of integrins and their cognate ligands.^32^ With the observed expression patterns, we hypothesized that cells with high *ITGA11* expression have high osteogenic potential, cells with high *NT5E* (encoding CD73) expression are deficient in osteoblast differentiation, and cells with high *CD151* expression neither favor one nor the other lineage. Using qPCR analysis in 33 primary cell strains, which have been previously characterized for their osteogenic differentiation potential,^21^ we found *ITGA11* mRNA expression in undifferentiated cells to be correlating with osteoblast differentiation while especially high *NT5E* mRNA levels were linked to poor osteogenic potential (Fig. 4e).

To validate whether the distinction of stromal cells by *ITGA11* and *NT5E* (encoding CD73) mRNA levels in vitro persists in vivo, we performed multiplex in situ hybridization on iliac crest bone biopsies (Fig. 4f). Using CD271 labeling, we found *ITGA11* and *CD151* to be enriched among stromal cells in human bone tissue (Fig. 4g). We further quantified the pairwise overlap of the three markers among all and CD271^+^ cells and found *ITGA11* and *NT5E* double positive cells to be depleted among stromal cells of the bone (Fig. 4h). Finally, in situ hybridization and scRNA-seq expression intensities displayed that high levels of *ITGA11* and *NT5E* transcripts were rare among stromal cells in vivo and in culture (Fig. 4i). These analyzes highlight, that *ITGA11* and *NT5E* transcripts mark different subsets of stromal cells in culture as well as in vivo.

**Fig. 5:**
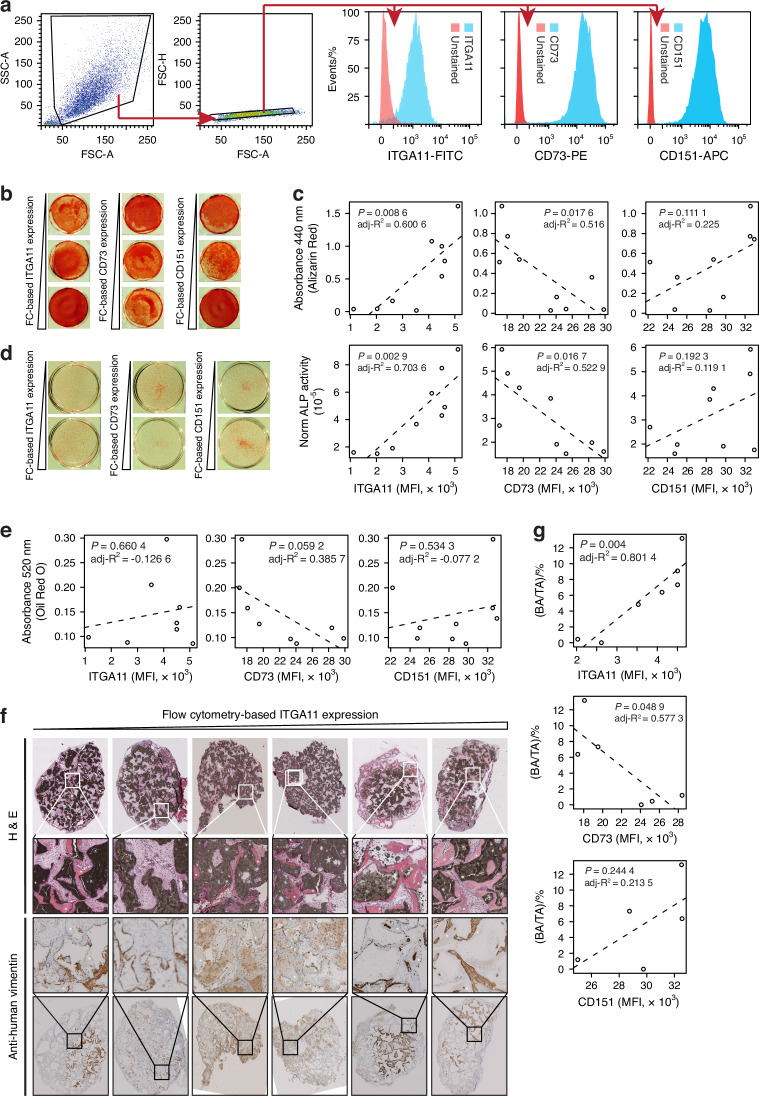
ITGA11 protein levels correlate with osteogenic potential in vitro and in vivo. **a** Cultured primary stromal cells from iliac crest bone marrow aspirates at OUH were subjected to flow cytometry. Gating strategy to remove debris and doublets and histograms showing staining for ITGA11, CD73, and CD151 versus unstained cells. **b** Primary cultures were subjected to flow cytometry-based quantification of ITGA11, CD73, or CD151 followed by osteogenic differentiation with Alizarin Red staining at day 14. **c** Scatter plot showing flow cytometry-based ITGA11, CD73, or CD151 protein levels in primary cultures prior to differentiation and quantification of their osteogenic potential based on matrix mineralization (Alizarin Red) or ALP activity. Statistics derived from linear regression. **d** Primary cultures were subjected to flow cytometry-based quantification of ITGA11, CD73, or CD151, followed by adipogenic differentiation with Oil Red O staining after 14 days. **e** Scatter plot showing flow cytometry-based ITGA11, CD73, or CD151 protein levels in primary cultures prior to differentiation and quantification of their adipogenic potential based on lipid droplet formation (Oil Red O). Statistics derived from linear regression. **f** H & E staining and anti-human vimentin staining of sectioned ectopic implants. ITGA11 expression in undifferentiated cells was measured by flow cytometry prior to implantation with hydroxyapatite in immunodeficient mice. Tissue was harvested after 8 weeks. Light red color in the H & E staining indicates newly formed bone, marked by white asterisk. Corresponding asterisks on anti-human vimentin staining highlights the presence of human cells. **g** Scatter plot quantifying flow cytometry derived expression levels of ITGA11, CD73, or CD151 in primary stromal cells and histological evaluation of bone formed by those cultures after 8 weeks of ectopic transplantation into immunodeficient mice. Statistics from linear regression. ITGA11 *n* = 7, CD73 *n* = 6, CD151 *n* = 5

Using fluorescently labeled antibodies, we determined ITGA11, CD73, and CD151 surface protein levels in primary human stromal cells and found almost all cells to be positive for the respective markers (Fig. 5a). Using absolute expression, we found ITGA11 surface protein levels to correlate with the osteogenic differentiation potential of the cells as determined by matrix mineralization and ALP activity (Fig. 5b, c). On the other hand, adipogenic differentiation potential was not linked to differences in ITGA11 protein levels (Fig. 5d, e). We observed an inverse relationship between CD73 cell expression levels and osteogenic (Fig. 5b, c) as well as adipogenic differentiation capacity (Fig. 5d, e). Using a xenograft model in immunodeficient mice,^33^ we next questioned whether surface molecule expression is predictive for the bone-forming potential of the cells. In line with the in vitro osteogenic capacity, cells with high ITGA11 levels exhibited higher bone-forming capacity in vivo (Fig. 5f, g) while primary cultures with high CD73 protein levels showed low bone-forming potential (Fig. 5g). As anticipated, we did not observe a correlation between CD151 protein expression levels and osteoblast differentiation capacity of the cells in vitro or in vivo.

Collectively, our scRNA-seq data set enabled us to identify two cell surface markers: ITGA11 and CD73, that based on expression demonstrate the capacity to predict osteogenic differentiation potential in vitro and in vivo, in a positive and negative manner, respectively. This finding is consistent with *ITGA11* expression being indicative of cells within cluster 0 and CD73 being representative of those within cluster 1.

### ITGA11-expressing stromal cells contain human SSCs

**Fig. 6:**
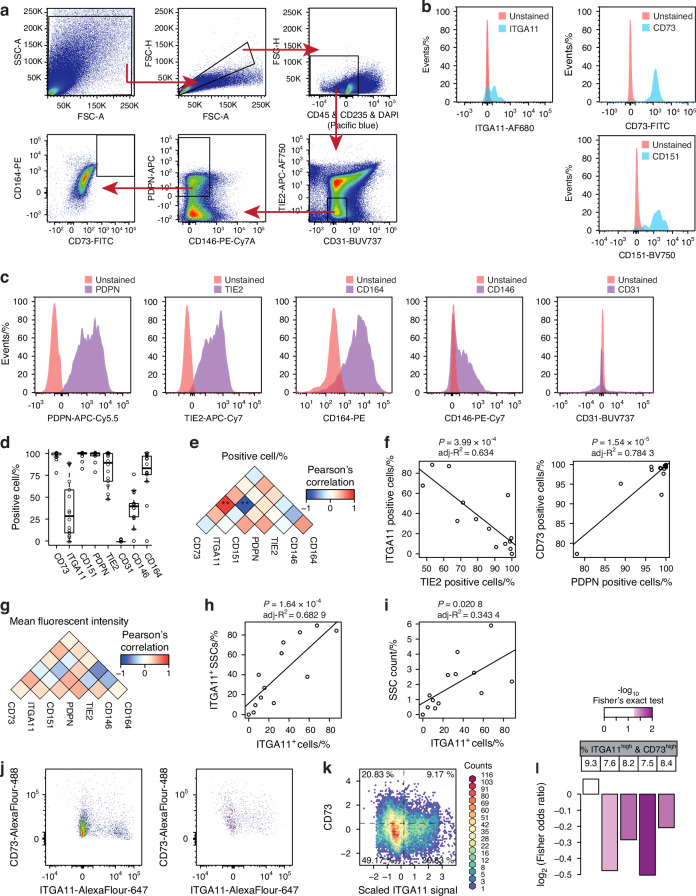
ITGA11 is expressed on skeletal stem cells. **a** Freshly isolated primary stromal cells from waste material of orthopedic surgeries at Stanford Hospital were stained for ITGA11, CD73, CD151, PDPN, TIE2, CD164, CD146, and CD31. Gating strategy for flow cytometry-based identification of skeletal stem cells (SSCs). **b** Histogram showing ITGA11, CD73, and CD151 staining compared to unstained controls on freshly isolated SSCs. **c** Cultured primary stromal cells were stained for ITGA11, CD73, CD151, PDPN, TIE2, CD164, CD146, and CD31 and subjected to flow cytometry. Histograms showing staining for PDPN, TIE2, CD164, CD146, and CD31 versus unstained cells. **d** Box plot quantifying marker-positive cells from **c**. **e** Heat map showing Pearson’s correlations between marker-positive cells in **d**. ** for *P* < 0.01 based on linear regression model. **f** Scatter plot illustrating significant associations from **e**. Statistics from linear regression. **g** Heat map showing Pearson’s correlations for marker expression levels (mean fluorescence intensity) on cells in **d**. **h** Scatter plot quantifying ITGA11-positive SSCs over total number of ITGA11-positive cells across 13 primary stromal cell cultures. Statistics from linear regression. **i** Scatter plot quantifying percentage of SSCs over total ITGA11-positive cells as in **h**. Statistics from linear regression. **j** Representative density scatter plots of ITGA11 and CD73 surface levels in ITGA11^+^, CD73^+^ cultured primary cells in **d**. **k** Density heat map showing the intra-donor scaled ITGA11 and CD73 staining intensities for five donors (at least 50% ITGA11^+^ cells) in **d**. Dashed lines indicate the arbitrary 70th-percentile intensity thresholds. **l** Bar plot showing enrichment of ITGA11 / CD73 double high cells (among 70th-percentile each) for donors pooled in **k**. Bar height indicates odds ratio, color encodes *P*-value for Fisher’s exact test. Percentage of double high cells are indicated

Chan et al. previously reported the identification of a human SSC with osteochondrogenic differentiation potential and no ability to form adipocytes.^13^ Due to the putative osteogenic capacity of *ITGA11*-expressing cells, we tested whether ITGA11 expression is linked to surface markers defining SSCs. Using the previously reported^13^ gating strategy (Fig. 6a), we first questioned whether ITGA11 is present on SSCs in vivo. Using cells freshly isolated from human bone pieces, we found human SSCs to express ITGA11 and CD151 in vivo (Fig. 6b). To quantify the relation of ITGA11 expression and SSC numbers, we next focused on established cultures of primary stromal cells. Here, we quantified expression of PDPN, TIE2, CD164, CD146, and CD31 together with ITGA11, CD73, and CD151 on primary stromal cell cultures (Fig. 6c, d). While almost all cultured cells were negative for CD31 and positive for CD73, CD151, and PDPN, there was a varying degree in the expression of ITGA11, TIE2, CD146, and CD164 (Fig. 6d). Focusing on percentage of positive cells, CD73^+^ cells correlated with PDPN^+^ cells, and ITGA11^+^ cells was inversely associated with TIE2^+^ cells (Fig. 6e, f). On the other hand, protein expression levels did not show any significant correlations between the examined markers across the donors (Fig. 6g). Gating cultured stromal cells for SSCs, we found the numbers of ITGA11^+^ stromal cells and ITGA11^+^ SSCs to be almost identical (Fig. 6h). In addition, the number of ITGA11^+^ stromal cells was positively associated with SSC abundance (Fig. 6i). However, while percentage of ITGA11^+^ stromal cells ranged from 0 to almost 90%, SSC numbers did not exceed 5% in primary stromal cultures (Fig. 6i). We therefore conclude that ITGA11 marks an osteogenic stromal cell population which contains SSCs to a minor degree and that the pro-osteogenic capacity is unlikely to be due to changes in SSC numbers. Finally, we questioned whether flow cytometry data could distinguish ITGA11^high^ from CD73^high^ cells, analogous to transcript-level differences observed by scRNA-seq in vitro and in situ hybridization in vivo. Focusing on ITGA11^+^, CD73^+^ cells, we found highly donor-dependent staining intensities for both markers (Fig. 6j). We therefore combined data in a scaled framework that revealed about 9% of cells to be high for ITGA11 and CD73 surface levels (Fig. 6k) using an arbitrary threshold of the 70th percentile, which we later used for sorting. Applying this threshold to individual donor samples demonstrated that ITGA11 and CD73 double high cells are depleted from the pool (Fig. 6l). Collectively, these data indicate that ITGA11 and CD73 are robust surface markers on cultured stromal cells and that their absolute expression levels are independent of each other.

### ITGA11-targeted cell sorting segregates stromal cells with high and low osteogenic potential

**Fig. 7:**
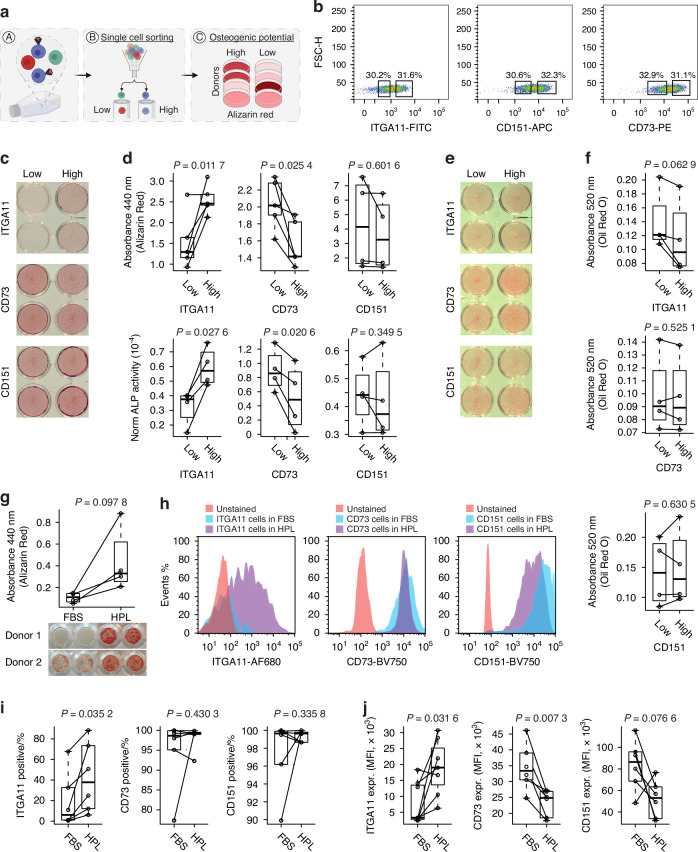
ITGA11 expression levels demarcate cells with high and low osteoblast potential. **a** Scheme illustrating the cell sorting strategy based on identified surface markers Ⓐ. Using FACS, cells of primary cultures were sorted based on high and low expression levels Ⓑ to compare the in vitro differentiation potential of both cultures derived from the same donor Ⓒ. Of note, we did not sort cells from the same donors for different markers. Generated in Biorender. **b** Cultured primary stromal cells derived from bone marrow aspirates at OUH were subjected to cell sorting based on ITGA11, CD73, and CD151 staining. Scatter plot showing the gating strategy for high- (top 30%) and low-expressing cells (bottom 30%). **c** Sorted cells as in Fig. 5a were subjected to osteogenic differentiation, and matrix mineralization was determined by Alizarin Red staining at day 14. **d** Sorted cells as in Fig. 7b were subjected to osteogenic differentiation. Box plot quantifying ALP activity and matrix mineralization (Alizarin Red) at day 7 and 14. High and low cells of individual donors are connected by lines. Paired *t*-test, *n* = 4. **e** Sorted cells as in Fig. 7b were subjected to adipogenic differentiation, and lipid droplet formation was determined by Oil Red O staining at day 14. **f** Sorted cells as in Fig. 7b were subjected to adipogenic differentiation. Box plot quantifying lipid droplet formation (Oil Red O) at day 14. High and low cells of individual donors are connected by lines. Paired *t*-test, *n* = 4. **g** Primary stromal cells from biological waste material of orthopedic surgeries at Stanford Hospital were cultured with FBS or HPL and subjected to osteogenic differentiation. Bar plot quantifying matrix mineralization (Alizarin Red) at day 14. Individual donors are represented by lines. Paired *t*-test, *n* = 4. **h** Histograms quantifying ITGA11, CD73, and CD151 staining on undifferentiated cells cultured in FBS or HPL as in Fig. 7g. Unstained cells served as control. **i** Box plot quantifying ITGA11, CD73, and CD151 positive cells by flow cytometry on cells cultured in FBS or HPL as in Fig. 7h. Individual donors are represented by lines. Paired *t*-test, *n* = 4. **j** Box plot quantifying ITGA11, CD73, and CD151 expression levels (mean fluorescence intensity) by flow cytometry on cells cultured in FBS or HPL as in Fig. 7h. Individual donors are represented by lines. Paired *t*-test, *n* = 4

Having identified ITGA11 as a candidate that potentially defines the proportion of stromal cells with high osteogenic potential, we wanted to sort primary stromal cultures into ITGA11 high and low expressing cells to test if the two populations derived from the same donor differ in their osteogenic potential (Fig. 7a). Using antibody labeling we prospectively isolated the top and bottom 30% of cells expressing ITGA11, CD73, or CD151 using FACS (Fig. 7b). In line with the ITGA11 expression associated osteogenic potential, we found ITGA11^high^ expressing cells to be more osteogenic than ITGA11^low^ expressing cells from the same donor (Fig. 7c, d). In contrast, CD73 sorting showed low osteogenic potential for CD73^high^ compared to CD73^low^ expressing cells (Fig. 7c, d). In general, there was little impact on adipogenic differentiation, with ITGA11^high^ expressing cells trending towards lower adipogenic potential compared to ITGA11^low^ expressing cells (Fig. 7e, f). Sorting cells based on CD151 expression did not affect the osteogenic or adipogenic differentiation potential of the cells. Importantly, sorting for either ITGA11 or CD73 could markedly separate low and high osteogenic cells from a single donor, irrespective of the given donor-to-donor variability in osteoblast differentiation potential.

Having established ITGA11^high^ cells to be pro-osteogenic, we questioned if culture conditions that enhance osteogenic potential, such as exposure to human platelet lysate (HPL) compared to fetal bovine serum (FBS),^34,35^ affect ITGA11 expression in stromal progenitors. HPL enhanced mineralizing capacity of cultured stromal cells (Fig. 7g), and this was associated with increased ITGA11 expression (Fig. 7h), both at the level of positive cell numbers (Fig. 7i) as well as absolute protein expression level (Fig. 7j). On the other hand, while the number of CD73^+^ or CD151^+^ cells did not change in primary stromal cell cultures incubated with FBS or HPL (Fig. 7i), absolute expression levels decreased in those exposed to HPL (Fig. 7j). Taken together, ITGA11 marks a subpopulation of stromal cells with high osteogenic differentiation potential, thus allowing the prospective separation into cells with either high or low osteogenic potential and its expression proxies osteogenesis-promoting culture conditions.

### ITGA11 marks but does not drive the expression levels of the osteogenic signature of cluster 0

**Fig. 8:**
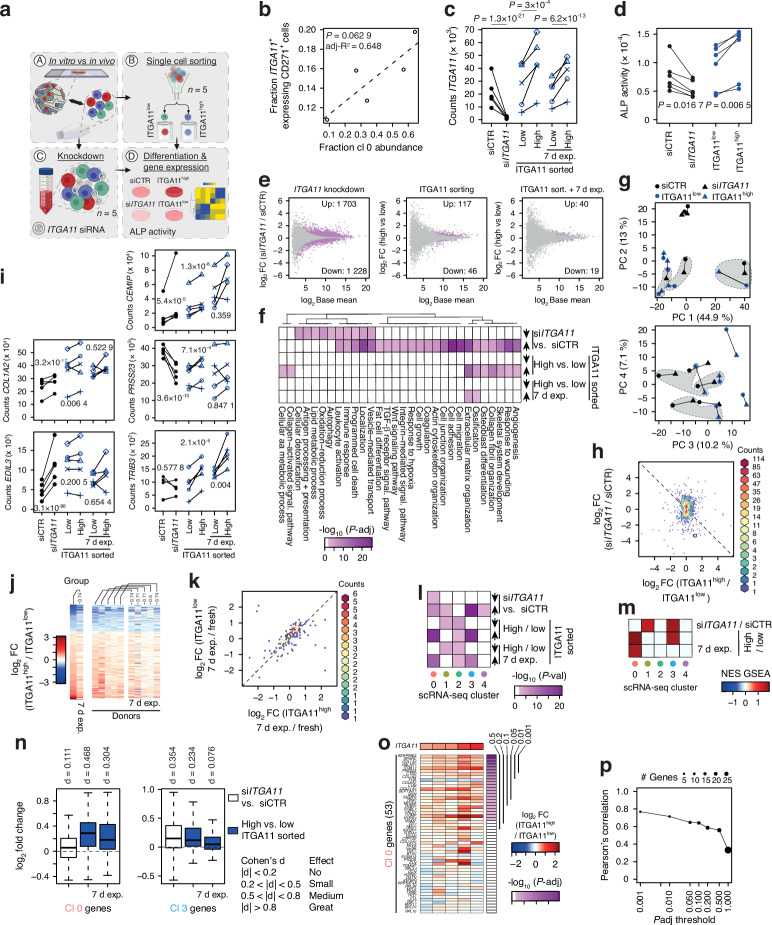
ITGA11 sorting interferes specifically with cluster 0 genes, distinct from ITGA11 knockdown. **a** Scheme illustrating the comparison of in vitro and in vivo heterogeneity of stromal cells Ⓐ as well as sorting for Ⓑ and knockdown of ITGA11 Ⓒ for comparative analysis at differentiation and gene expression levels Ⓓ. Generated in Biorender. **b** Scatter plot of cluster 0 abundance in scRNA-seq data and percentage of CD271^+^ cells expressing *ITGA11* from iliac crest bone biopsies. In situ hybridization of *ITGA11* (TxR) and CD271 immune staining (Cy3) on biopsies from 5 participants included in the scRNA-seq data. Statistics from linear regression. **c** RNA-seq based counts of *ITGA11* in primary stromal cells upon *ITGA11* knockdown (black) or sorting for ITGA11 high and low expressing cells (blue) before (middle) and after 7 days of expansion (right). Shapes indicate donors, lines connect donor samples across conditions. DESeq2 adjusted *P*-values. **d** ALP activity after 7 days of osteogenic differentiation in primary cells with knockdown of *ITGA11* (black) or sorted for high and low ITGA11 expression (blue). Lines connect donor samples across conditions. *P*-value from paired *t*-test. **e** MA-plots for differential gene expression analysis of primary cells described in Fig. 8c. Genes with DESeq2 adjusted *P*-values < 0.05 in pink. **f** Gene ontology enrichment for gene groups shown in Fig. 8e. Heat map showing the Benjamini–Hochberg adjusted *P*-value retrieved from go-seq (cutoff *P* < 0.01). **g** PCA-plots showing variance in gene expression in primary cells upon *ITGA11* knockdown or sorted for low and high ITGA11 expression. Lines connect donor samples across conditions. Grey fields indicate donor samples subjected to sorting and knockdown. **h** Density scatter plot showing log-2 fold changes in gene expression upon *ITGA11* knockdown and sorting for low and high expression of ITGA11. Circle indicates *ITGA11*. **i** RNA-seq based counts of *CEMIP, COL1A2, PRSS23, EDIL3, and TRIB3* in primary stromal cells described in Fig. 8c. Adjusted *P*-values from DESeq2. **j** Heatmap showing log-2 fold changes in gene expression upon sorting for low and high expression of ITGA11 prior to and after 7 days of expansion at the group and donor level. Lines link donors across time points, Pearson’s correlations are indicated. **k** Density scatter plot showing log-2 fold changes during 7 days of expansion in ITGA11^high^ (*x*-axis) and ITGA11^low^ (*y*-axis) populations for genes selected in Fig. 8j. Circle indicates *ITGA11*. **l** Enrichment between cluster-specific genes (enriched markers in Fig. 2b) and gene groups shown in Fig. 8e. *P*-value from hypergeometric test (cutoff *P* < 0.01). **m** Gene set enrichment analysis using cluster specific gene signatures (enriched markers in Fig. 2b) and gene ranking based on contrast in Fig. 8e. Heat map showing fgsea derived normalized enrichment score (cutoff *P*-adj < 0.05). **n** Box plots quantifying log-2 fold changes of cluster 0 and 3 gene signatures (enriched markers in Fig. 2b) for conditions contrasted in Fig. 8e. Cohen d effect sizes to random genes. **o** Heat map quantifying log-2 fold changes for cluster 0 genes (enriched markers in Fig. 2b) upon sorting for low and high ITGA11 expression. Donors were ordered by differences in *ITGA11* expression, genes were ordered by DESeq2-adjusted *P*-values. **p** Pearson’s correlation between changes in *ITGA11* expression and the average changes in selected cluster 0 genes from Fig. 8o. Circle size indicates number of genes

Having identified ITGA11 as a discriminator for the osteogenic potential in vitro, we wanted to test whether ITGA11-based heterogeneity in culture persists in vivo and whether ITGA11 actively contributes to the osteogenic gene signature in cluster 0 comparing loss of function experiments with cells right after sorting for ITGA11 levels as well as after 7 days of expansion (Fig. 8a). Based on bone biopsies from five subjects that were included in our scRNA-seq data, we could confirm that the abundance of cluster 0 cells in scRNA-seq strongly correlates with the fraction of *ITGA11* expressing CD271^+^ stromal cells (Fig. 8b), suggesting that this transcriptionally defined population corresponds to the ITGA11^+^, CD271^+^ compartment in vivo. Statistical significance was limited by the small number of donors.

Using siRNA mediated knockdown and ITGA11 sorting, we showed that loss of *ITGA11* expression (Fig. 8c) decreases osteogenic activity of primary stromal cells (Fig. 8d). Using bulk RNA-seq analysis we identified many more genes to be affected by loss of *ITGA11* expression compared to cells sorted for ITGA11 surface levels (Fig. 8e). Accordingly, we found many more biological functions affected by knockdown of compared to sorting for ITGA11 (Fig. 8f). Interestingly, genes related to osteoblast, matrix, and skeletal function were affected by both perturbations but opposingly regulated, i.e., elevated expression upon loss of *ITGA11* and in ITGA11^high^ cells. To follow up, we performed unbiased principal component analysis and surprisingly identified different trajectories for knockdown of and sorting for ITGA11 (Fig. 8g), even for samples from the same donor. In line, differential gene expression for *ITGA11* knockdown and sorting for ITGA11 did not align (Fig. 8h), manifesting that loss of ITGA11 signaling is molecularly distinct from sorting cells with high and low levels of ITGA11 surface molecules. With focus on selected examples (Fig. 8i), we identified rare examples like cluster 0 gene *PRSS23* which followed the expression levels of *ITGA11* (Fig. 8c), genes like *EDIL3* and *TRIB3* solely affected by one perturbation, and cluster 0 gene *COL1A2* and the suppressor of osteoblast differentiation *CEMIP*^36^ with opposing regulation.

Next, we examined the changes induced by ITGA11 sorting and found that, although attenuated after 7 days of subculturing, their overall direction was robustly maintained at the group level as well as across donors (Fig. 8j). While the ITGA11^high^ and ITGA11^low^ populations converge over time, we unexpectedly found, that ITGA11 sorting-sensitive genes changed strikingly similar in both populations overriding the differences imposed by sorting (Fig. 8k).

Finally, with respect to ITGA11 sorting demarcating the osteogenic gene signature, both overlap (Fig. 8l) and gene set enrichment analysis (Fig. 8m) highlighted ITGA11^high^ cells to be strongly enriched for cluster 0 genes. This was further supported as cluster 0 genes were upregulated comparing ITGA11^high^ to ITGA11^low^ cells but not affected by knockdown of ITGA11 (Fig. 8n). This demonstrates that ITGA11 sorting, but not interference with ITGA11 signaling, modulates the cluster 0 gene signature. Moreover, donor-to-donor differences in the expression of cluster 0 genes closely mirrored the corresponding variation in ITGA11 mRNA levels (Fig. 8o, p), indicating that ITGA11 not only marks this osteogenic program but also quantitatively reflects its activity. Taken together, these data strongly support an intrinsic osteogenic cell-to-cell heterogeneity which can be traced by the expression of ITGA11 but does not dependent on the integrity of ITGA11 signaling.

## Discussion

Our study addressed the heterogeneity among stromal cells in culture as well as the donor-to-donor variability in subpopulation composition and how both are linked to osteogenic function and commitment. We report cultured stromal cells to be heterogeneous and that subpopulation-specific gene signatures are, first, implicated in bone-related pathways and skeletal genetic traits and, second, can be projected on individuals with inter-donor variation in subpopulation composition. The abundance of a particular subpopulation, marked but not driven by the expression of *ITGA11* and genes involved in extracellular matrix organization, translates to the osteogenic potential of the pool of primary cells and allows for the prospective isolation of cells with high and low osteogenic potential from a single donor.

### Heterogeneity among cultured stromal cells

While recent single-cell RNA-seq data of developing human bones have outlined the presence of stromal cells with distinct transcriptional profiles related to spatial mapping and lineage preferences,^19,20^ cultured cells are devoid of a complex spatial and temporal context. Nevertheless, single-cell RNA-seq approaches have been shown to identify transcriptional heterogeneity among cultured stromal cells,^37–40^ commonly identifying a proliferating population as well as cells with putative elevated stemness, an inflammatory signature, or signs of lineage commitment. Immune and extracellular matrix processes have been postulated to mainly drive heterogeneity among cultured stromal cells from various tissue sources.^38^ In our data, subpopulation identity was largely not linked to immune pathways; instead, genes related to matrix processes as well as multiple signaling pathways, metabolism, and an angiogenic response were key in distinguishing the cell populations. Interestingly, part of the heterogeneity in our data was driven by NRF2-mediated senescence gene signature, related to Gao et al. reporting a varying degree of cellular senescence in cultured stromal cells due to tissue source or donor age.^37^ While linking subpopulations through lineage hierarchy is tempting, we did not perform trajectory analysis as evidence for a functional hierarchy among cultured cells (out of spatial and niche context) is limited. Instead, we focused on functional annotation of the clusters and highlighted for the first time that subpopulation-specific genes are implicated in bone-specific pathways, associated with genetic traits of bone mass in humans and mice, and are subject to transcriptional remodeling during lineage specification. Surprisingly, the latter was not linked with an adipogenic or osteogenic preference and therefore contradicts the hypothesis of stromal cells being populated by clonal and lineage-restricted cells.^41,42^ While the source of heterogeneity might be due to intrinsic signaling events, epigenetic programs, and stochastic variations, it remains to be determined what drives and maintains the abundance of individual subpopulations, as these are subject to expansion in vitro. Despite we could link the abundancies of an *ITGA11*-marked stromal cell population in vitro and in vivo based on paired primary cultures and iliac crest bone biopsies, it needs to be determined whether repeated sampling from the same donor will yield a similar subpopulation distribution.

### Heterogeneity as a predictor for the donor-specific osteogenic potential

A high inter-donor variability in osteogenic and/or bone regenerative capacity of in vitro expanded stromal cells has been reported in pre-clinical culture or transplantation assays^21,43^ and hypothesized to be present in the cellular products used in the clinical trials.^44–46^ This variation might limit the use of stromal cells for regenerative therapies as it may lead to unpredictable clinical outcomes. Our laboratory has previously shown that cellular and nuclear morphology features^21^ as well as clinical parameters^22^ were able to predict the osteogenic differentiation capacity of primary cells in vitro. For example, the number of CD146^+^ cells was positively predicting osteogenic potential and could explain up to 46% of the inter-donor variation, while adding sex, vitamin D supplementation, number of ALP^+^ cells, number of CD14^+^ cells, and osteoporosis history accounted for up to 55%.^22^ Thus, molecular features and donor characteristics affect the function of cultured stromal cells. Therefore, we sought to identify gene signatures that demarcate molecularly defined subpopulations and that allow the prospective isolation of high and low osteogenic cells from an individual donor.

Clearly, cultured stromal cells are heterogeneous as also reported by Wang et al. based on scRNA-seq of bone-marrow (BMSC), adipose tissue (AMSC), umbilical-cord (UMSC) and dermal tissue (DMSC) derived stromal cells.^38^ While some identified cell clusters were specific to tissue of origin, their abundancies varied amongst donors, highlighting cellular heterogeneity among stromal cells with tissue priming effects and donor-to-donor variation. Here, we demonstrate that some individual cultures were indeed highly donor-specific, while the majority of cultures revealed the presence of multiple molecularly defined subpopulations. Using the power of 26 scRNA-seq libraries from primary cultures, donor-to-donor differences in osteogenic commitment were strongly linked to the distribution of subpopulations. In addition to the predictive power of individual subpopulations, combining multiple ones yielded stronger associations with osteogenic differentiation (data not shown), indicating that additional markers for combinatorial positive and negative selection could further improve the definition of highly osteogenic cells.

### Osteoprogenitor cell surface markers and donor attributes

The current definition of MSCs proposed by the International Society for Cellular Therapy is ineffective in distinguishing between multipotent stromal cells, committed and more restricted progenitors, as well as highly proliferative cells, and can therefore not be used to prospectively isolate populations with specific phenotypes and functions.^47^ Indeed, surface marker expression has been used to describe human stromal cell subpopulations^48^ with enhanced osteogenic potential, such as comparing CD90^+^ with CD90^−^ cells,^49^ or the role of CD34^50^ or CD146.^51^ However, it is still debatable whether expression of those markers is qualitatively linked to osteoblastic differentiation or characterizes a minimal requirement of a stromal cell phenotype. As such, expression of CD90 is stable throughout in vitro expansion despite a decline in differentiation potency.^52^ Therefore, primary and secondary tier markers have been suggested, e.g., CD73 and CD105, with their expression being predictive for differentiation competent cells,^53^ as well as CD34, CD106, and PODXL, with their expression being linked to differentiation potential, age, and proliferative capacity of the cells.^54–57^ In line, we reported that cultured cells do uniformly express CD44, CD73, CD105, and CD90, while expression of CD146, CD271, CD34, CD362, and ALP varied tremendously between individual donors.^21,22^ Interestingly, we found differences in the expression of both primary and secondary tier markers across the 5 subpopulations, exemplified by the heterogeneity in *NT5E* transcript levels in vitro and in vivo. Importantly, as all cells in our study express CD73 surface molecules in vitro, we do not distinguish CD73^-^ from CD73^+^ cells, nor do we interfere with CD73 or adenosine signaling, which has well-documented osteogenic activity on human cells^58^ and mice in vivo.^59,60^ Instead, our findings—particularly those based on interference with ITGA11— suggest that variation in marker transcript levels reflects a consequence, rather than a driver, of osteogenic state. In this context, the observation that CD73^low^ cells exhibited greater osteogenic potential than CD73^high^ cells in vitro, as well as for ALP^-^ cells of human adipose tissue,^61^ supports the notion that CD73 abundance does not linearly correspond to osteogenic priming and that *NT5E* expression levels reflect a functional heterogeneity rather than a defined osteoprogenitor identity.

### ITGA11

Here, we reported that *ITGA11* expression marks a subpopulation of stromal progenitors with osteogenic differentiation potential and that ITGA11 surface molecule levels allow prediction of osteogenic capacity and isolation of highly osteogenic cells. This questions whether ITGA11 only marks an osteoprogenitor cell population or whether it is functionally involved in the differentiation process. ITGA11 is an osteolectin receptor with nanomolar binding affinity, and genetic studies in mice showed that ablation of the receptor or ligand diminishes bone formation.^30,62^ This indicates that ITGA11 is a qualitative marker of osteoblastic cell function as its activation leads to increased WNT signaling and osteoblast differentiation in vitro.^30^ *ITGA11* exhibits a 20-fold higher expression in differentiation-competent stromal cells compared to fibroblasts,^47^ and higher levels in bone marrow-derived stromal cells compared to adipose,^63^ lung,^64^ or umbilical vein-derived ones.^65^ In line with our scRNA-seq data, *ITGA11* expression was found on leptin receptor-positive stromal cells.^30^ Still, *ITGA11* expression is not restricted to progenitor cells, as it has been quantified with stable^27,30,65^ or even increased^66^ expression upon osteogenic differentiation in vitro. Interestingly, osteogenesis-enhancing culture conditions such as hypoxia^67,68^ or TGF-β treatment^69^ are associated with higher *ITGA11* expression,^64,70^ corroborating our findings of increased *ITGA11 gene* expression in cultures supplemented with human platelet lysate. Importantly, these observations indicate a pivotal role of ITGA11 in osteogenic commitment; however, concerning the heterogeneity of stromal cells in culture, ITGA11 reflects a molecular signature comprising 53 genes that likely mediate osteogenic function in a coordinated manner. Importantly, while both ITGA11^low^ and *ITGA11* knockdown cells exhibited diminished osteogenic potential compared to ITGA11^high^ and control cells, respectively, the expression of that osteogenic gene signature was only decreased by sorting, underlining the distinction between the effect of a signaling pathway on the one side and the intrinsic transcriptional networks on the other side. Clearly, donor-specific variance in the osteogenic commitment persisted among ITGA11 high- and low-expressing cells, making ITGA11 an attractive tool in terms of versatility, but also highlighting the importance of lifestyle and donor attributes in the search for cell products fostering bone regeneration in a highly reproducible manner. The fact that culture conditions can affect ITGA11 levels and osteogenic capacity makes it attractive to identify signaling cues that control ITGA11^high^ cell abundance. And in turn, exploration of whether conditions that hamper bone formation in vivo, such as aging^71^ or glucocorticoid exposure,^72,73^ affect the abundance of ITGA11 high-expressing stromal cells would support new treatment strategies to elevate ITGA11^high^ cell numbers to restore or maintain bone remodeling to combat bone loss.

### Limitations

Our study designs included a variety of stromal cell sources, from bone waste products of orthopedic surgery as well as marrow aspirates, across male and female, various ages, and subjects with and without osteoporosis. While this broad spectrum of primary samples supports the versatility of our *ITGA11*-marked cell signature for osteogenic properties, the distribution of samples across those conditions does not allow statistical analysis of whether the abundance of this subpopulation is affected by sex, age, or osteoporosis. With the high demand of cells needed to perform reliable outcomes in ectopic transplantation assays, we are currently limited to comparing the in vivo bone-forming potential of ITGA11^high^ versus ITGA11^low^ cells as well unsorted ones from primary cultures. Alternatively, human bone biopsies suitable for dynamic histomorphometry should be tested for *ITGA11* in situ hybridization to overlay active bone formation with *ITGA11* mRNA levels in vivo.

## Materials and methods

### Isolation of primary bone marrow-derived stromal cells and cell culturing

Stromal cells were isolated from bone marrow aspirates or biological waste material from orthopedic surgeries at Odense University Hospital or Stanford Hospital. At Odense University Hospital, bone marrow aspirates were collected from the iliac crest or the lower extremities of donors undergoing surgeries at the Department of Orthopedics and Traumatology. Clotting was prevented by mixing 10 to 15 mL aspirate with 10% Heparin (748590, Amigros) in minimum essential media (MEM, 32561-094, Invitrogen). Stromal cells were cultured from the mononuclear cell population obtained through gradient centrifugation using Lymphoprep (170546, ILS Danmark) and subsequent plastic adherence to tissue culture flasks as previously described.^74^ Cells were seeded in MEM media supplemented with 10% fetal bovine serum (FBS, 16000044, Life Technologies) and 1% penicillin/streptomycin (P/S, 15140-130, Invitrogen), under standard culture conditions (37 °C with 5% CO_2_ and 95% humidity). After a week, when the first adherent cells became visible, media was refreshed three times a week until reaching approximately 80% confluence. At this stage, cells were trypsinized using 0.05% Trypsin-EDTA (25300062, Thermo Fisher Scientific) and passaged for further expansion. At Stanford Hospital, femoral heads were provided as biological waste material from orthopedic surgeries. Bone marrow was scraped off bone specimens and minced with sterilized razor blades, followed by suspension in digestion buffer containing 3 mg/mL type II collagenase (C6885, Sigma-Aldrich) and 100 U/mL DNase I (LS002007, Worthington Biochemical Corporation). After 40-min digestion at 37 °C, cells were filtered through a 70 μm nylon mesh and centrifuged at 200 × *g* for 5 min at 4 °C. The supernatant was discarded, and cells were (1) resuspended in culture media (MEM supplemented with 1% P/S and either 10% Human Platelet-derived Lysate (HPL, 06962, Stemcell Technologies) or 10% FBS for subsequent expansion or (2) resuspended in FACS buffer (2% FBS in phosphate-buffered saline devoid of Ca^2+^ and Mg^2+^ (PBS, 14190-169, Invitrogen)) for subsequent flow cytometry or sorting.

### In vitro cell differentiation

#### Osteoblast differentiation

Cells were seeded at a cell density of 20 000 cells/cm^2^, achieving approximately 80% confluency the day after, and then expanded for 72 h in MEM media supplemented with 10% FBS or HPL, 1% P/S. Following this period, the culture medium was substituted with osteoblast induction medium, comprised of MEM supplemented with 1% P/S, 5 mmol/L β-glycerophosphate (50020-100, Sigma-Aldrich), 10 nmol/L dexamethasone (D4902, Sigma-Aldrich), 50 µg/mL 2-phosphate ascorbic acid (A4544, Sigma-Aldrich), 10 nmol/L 1,25-vitamin D_3_ (D1530, Sigma-Aldrich) and either 10% FBS or 10% HPL. The induction medium was refreshed every 2–3 days up to 14 days of differentiation.

#### Adipocyte differentiation

Cells were seeded at a cell density of 30 000 cells/cm^2^, achieving approximately 100% confluency the day after, and then maintained for 72 h in MEM media supplemented with 10% FBS or HPL, 1% P/S. Following this period, the culture medium was substituted with adipocyte induction medium, comprised of Dulbecco’s modified Eagle’s medium (DMEM, 41965062, Thermo Fisher Scientific) supplemented with 1% P/S, 100 nmol/L dexamethasone, 3 µg/mL insulin (I9278, Sigma-Aldrich), 1 μmol/L rosiglitazone (BRL, 71740, Cayman Chemicals), 225 μmol/L 3-isobutyl-1-methylxanthine (IBMX, I5879-5G, Sigma-Aldrich) and either 10% FBS or 10% HPL. The induction medium was refreshed every 2–3 days up to 14 days of differentiation.

### Differentiation assays

#### Alkaline phosphatase (ALP) activity

Cells were seeded in 96-well plates, and ALP activity was assessed 7 days after induction of osteoblastic differentiation ALP activity was assessed. First, cell viability was evaluated using the CellTiter-Blue assay.^75^ Differentiation media was substituted with a mixture of CellTiter-Blue (208657 (G8081), ILS Danmark) and culture media at a ratio of 1:5, followed by an incubation at 37 °C for 1 h. Fluorescence intensity (excitation at 579 nm and emission at 584 nm) was recorded using a FLUOstar Omega plate reader. Second, to measure ALP activity, cells underwent two washes with tris-buffered saline (TBS, Lab44416.5000, Bie & Berntsen) at a pH of 7.5, followed by fixation in a solution of 3.7% formaldehyde (F1635, Sigma-Aldrich) and 90% ethanol (51976, Sigma-Aldrich) for 30 s. Subsequently, cells were incubated with 1 mg/mL of P-nitrophenylphosphate (dissolved in 50 mmol/L NaHCO_3_ and 1 mmol/L MgCl_2_ at pH=9.6, 71768-5G, Sigma-Aldrich) for 20 min at 37 °C. Absorbance was recorded at 405 nm, and ALP activity was normalized to cell viability.

#### Alizarin Red staining and quantification

Matrix mineralization was quantified using Alizarin Red staining. The process involved washing 14-day differentiated cells with PBS, followed by fixation in 77% ice-cold ethanol at −20 °C for 1 h and subsequent rinsing with H_2_O. Fixed cells were then stained with a solution of 40 mmol/L Alizarin Red dye (A5533-25G, Sigma-Aldrich) in distilled H_2_O (pH 4.2) for 10 min with agitation at room temperature (RT), and afterwards, cells were briefly rinsed with PBS. The stained samples were scanned and imaged at 4× magnification using an Olympus IX50 microscope. For quantification of Alizarin Red staining, dye was extracted using Alizarin Red extraction media (20% methanol (65548, Sigma-Aldrich), 10% acetic acid (20302236, VWR), and 70% H_2_O) for 10 min at RT with agitation. Absorbance of the solution was measured at both 450 nm and 570 nm.

#### Oil Red’O staining and quantification

To assess lipid accumulation, Oil Red O staining was employed. After 14 days of differentiation, cells were rinsed with PBS, fixed with 4% paraformaldehyde (PFA, D383004, Hounisen) in PBS at room temperature (RT) for 10 min, and subsequently washed with PBS. Fixed cells were stained with a solution consisting of 3 mg/mL Oil Red O (O0625, Sigma-Aldrich) dissolved in 100% isopropanol (I9516, Sigma-Aldrich) mixed with H_2_O at a ratio of 3:2 for 1 h at RT, followed by a rinse with H_2_O. Images of lipid droplets were captured at a 10× magnification using an Olympus IX50 microscope. To quantify Oil Red O staining, the dye was extracted by adding 100% isopropanol to the cells. After incubation for 10 min at RT with agitation, absorbance of the solution was measured at 500 nm.

### siRNA transfection

Primary cells at passage 2 or 3 were subjected to reverse transfection 3 days prior to differentiation (day -3), using a final concentration of 30 nmol/L siRNA (Thermo Fisher: ITGA11: #4390824, Negative Control: #4390843), 0.15% lipofectamin 2000 and 140 000 cells per mL using alphaMEM without additives. For example, to transfect a cell for a single 12-well, 1.5 µL of 20 μmol/L siRNA stock was mixed with 150 µL alphaMEM, 1.5 µL lipofectamin 200 was mixed with 150 µL alphaMEM, and both mixtures were united after 5 min incubation. After additional 20 min incubation, 140 000 cells in 0.7 mL were added and 1 mL of cell-transfection-mix was plated to a 12-well, yielding 40 000 cells/cm^2^. Transfection mix was replaced after 4 h with growth media, and after 3 days differentiation was induced (day 0). Knockdown efficiency was measured at the mRNA level by qRT-PCR.

### Ectopic bone formation

Assessment of bone-forming capacity using an in vivo transplantation assay has been previously described in detail.^33^ Shortly, we used 500 000 primary stromal cells per implant and performed 2 implants per primary culture. Implants were harvested after 8 weeks and fixed in 4% PFA for 24 h followed by decalcification 0.5 mol/L sodium format for 72 h. Implants were paraffine-embedded and sectioned at three different depths (100 µm apart). H&E stained sections were quantified for bone using ImageJ by marking areas of bone normalized to the total area of the implant. Average of the three depths, as well as the two implants, was used to report BV/TV per donor culture. As a deviation from the published protocol, NOD.Cg-*Prkdc*^*scid*^ *Il2rg*^*tm1Sug*^/JicTac mice have been used instead of NOD/LtSz-*Prkdc*^scid^ mice. Ethical approval was given (License 2022-15-0201-01225).

### Real-time PCR (qRT-PCR)

RNA was extracted from cultured progenitor cells using TRIzol (15596018, Invitrogen) and isolated with the QIAGEN RNeasy mini kit. The extracted RNA was then converted into cDNA using the RevertAid H Minus First Strand cDNA Synthesis Kit from Thermo Scientific^TM^. For quantitative Real-time PCR, Fast SYBR Green Master Mix (4385612, Applied Biosystems) and specific primers for *ITGA11*, *CD151* or *CD73* from Pentabase A/S were prepared. This mixture was then analyzed using the Applied Biosystems 7500 Real-Time PCR System. *Alpha-tubulin* or *GAPDH* served as reference housekeeping genes for these experiments. Primer sequences for *ITGA11* (For: GCCTGGTGGTGGCCTG; Rev: CCCACGACCAGCCACTTATT), *CD151* (For: CCTAGAGTCCTGGGGAGCTT; Rev: GGCCAGCCAGCCAGAAG), *CD73* (For: ACCTGATTTGTGATGCAATGATTA; Rev: TGGATTCCATTGTTGCGTTCA), *Alpha-tubulin* (For: GAGGCTGACGCAGAATGCA; Rev: TCTGTGGCAATCCGGTTCA) and *GAPDH* (For: GGAGCGAGATCCCTCCAAAAT; Rev: GGCTGTTGTCATACTTCTCATGG) were used.

### Single cell RNA-seq

Primary stromal cell cultures of 26 donors were subjected to single-cell RNA-sequencing. Cultured cells at passage 1–3 were trypsinized upon reaching 80%–90% confluence, spun down at 200 × *g*, resuspended in culture medium and separated using a 40-µm mesh. Then, cells were washed three times using PBS with 0.04% non-acetylated BSA (B6917, Sigma-Aldrich). Cell stock concentration of 0.7 × 10^6^ cell/mL was adjusted and flushed through a 40-µm mesh to be used as input material. Library preparation was done according to manufacturer’s guidelines (10× Genomics, version 3.1) from 10 000 cells of input material. Libraries were sequenced on a Nova seq, and raw reads were processed using the zUMI pipeline^76^ to quantify gene expression at the intron and exon level using the human genome GRCh38 for alignment. Subsequently, each single-cell data set underwent step-by-step filtration to retain high-quality droplets. Empty droplets were removed using emptyDrops,^77^ cells with mitochondrial percentage above 10% and gene count below 200 were removed, outlier detection was determined based on principal component analysis of quality matrices using Scater,^78^ doublet scores were calculated using scran,^79^ and cells were clustered in Seurat^80^ at high resolution to detect and remove clusters that separated based on doublet score and low feature or UMI number. Finally, data sets were merged in Seurat using Harmony^81^ to correct for batch effects. This process resulted in a refined gene count matrix, encompassing 136 014 cells. Differentially expressed genes were determined in Seurat by comparing the cells of one cluster against all other cells (enriched markers) or against the cells of each cluster individually (exclusive markers).

### Bulk RNA-seq

RNA was extracted from cultured cells using Trizol combined with Econo Spin columns (Epoch Life Sciences) and RNA-seq libraries were prepared according to manufacturer’s instructions (TruSeq 2, Illumina) using 1 µg RNA for preparation of cDNA. RNA-seq reads were aligned to GRCh38 (hg38) genome using STAR^82^ and default settings. Bam files were used to quantify tag distribution at gene bodies using featureCounts from the Subread package.^83^ Normalization and differential expression analysis were performed using DEseq2^84^ comparing treatment conditions (siITGA11 vs. siCTR, ITGA11^high^ vs ITGA11^low^ before and after 7 days of expansion) and including donor information in the model.

### Enrichment analysis of cluster-specific gene signatures

Cluster-enriched or -exclusive genes were subjected to open-source WikiPathway^85^ and Gene Ontology^86^ analysis using GOseq^87^ to predict subpopulation-specific biological functions.

GWAS summary statistics for estimated bone mineral density^26^ were retrieved from http://www.gefos.org/?q=content/data-release-2018. SNPs were linked to genes using transcription start sites annotated in the R package TxDb.Hsapiens.UCSC.hg19.knownGene and the R package Genomic Ranges^88^ with a variable window from 50 to 2 500 kb. Enrichment was calculated using a Pearson’s chi-square test, which compares the ratio of significant (*P* < 5 × 10^−8^) to nonsignificant SNPs (*P* > 5 × 10^−8^) for the enriched genes of a particular cluster as well as all detected genes from the scRNA-seq analysis.

Data on mouse knockout phenotypes were collected from the International Mouse Phenotyping Consortia (http://ftp.ebi.ac.uk/pub/databases/impc/all-data-releases/latest/results/). We used a hypergeometric test to perform enrichment analysis for the overlap between cluster-specific genes and human orthologs to genes linked to an X-ray-defined phenotype in knockout mouse models.

Processed gene expression data for human bone marrow-derived and telomerase-immortalised stromal cells (hBM-MSC-TERT4 cells) during osteoblast and adipocyte differentiation^27^ were downloaded from the GEO datasets GSE113253. Fold changes, adjusted *P*-values, and transformed expression data for day 0 and day 7 of differentiation were extracted. Principal component analysis was done on expression data of hBMSC-TERT4 cells restricted to cluster-specific gene signatures. A hypergeometric test was used to calculate enrichments for the overlap between cluster-specific genes and genes with differential expression at day 7 (FDR < 0.01) of osteoblast and adipocyte differentiation was calculated using a hypergeometric test.

Enrichment between cluster signatures and bulk-RNA-seq of ITGA11 knockdown or sorted cells was done using a hypergeometric test or gene set enrichment analysis using the fgsea^89^ in R.

### Flow cytometry and fluorescence-activated cell sorting

Trypsinized cultured stromal cells or cells freshly retrieved from femur heads were centrifuged at 200 × *g* for 5 min and then resuspended in FACS buffer. A portion of the cells was kept unstained for reference, while the remaining cells were either stained to target individual surface molecules per sample using directly conjugated primary antibodies or unconjugated primary antibodies followed by adding fluorophore-conjugated secondary antibodies. As primary antibodies we used unconjugated ITGA11 (0519-100/ITGA11-203E3, Nanotools), APC-CD151 (300406, BioLegend) and PE-CD73 (550257, BD Biosciences) and FITC-conjugated anti-mouse immunoglobulin (F0261, Dako Denmark A/S) as secondary antibody. To evaluate SSCs, cells were stained using a cocktail composed of the following primary antibodies BV750-CD151 (747487, BD Biosciences), AF680-ITGA11 (self-made conjugate of 0519-100/ITGA11-203E3, Nanotools and A20108, Thermo Fisher Scientific), BUV737-CD31 (748320, BD Biosciences), Pacific Blue-CD45 (304029, BioLegend), Pacific Blue-CD235ab (306612, BioLegend), Biotin-Tie2 (334204, BioLegend), PE-Cy7A-CD146 (342010, BioLegend), APC-PDPN (17-9381-42, Thermo Fisher Scientific), PE-CD164 (324808, BioLegend) and FITC-CD73 (344016, BioLegend) followed by co-staining with streptavidin (APC-Alexa Fluor 750, SA1027, Thermo Fisher Scientific). Upon staining cells with one or multiple primary antibodies, cells underwent incubation at 4 °C for 45 min, followed by three washes with FACS buffer. Subsequently, cells were stained with secondary antibodies or streptavidin at 4 °C for 45 min and washed three times with FACS buffer. Prepared cells were transferred through a 70 µm cell strainer into a 5 mL Falcon round-bottom tube, then subjected either to Flow Cytometry on a BD LSRII or BD FACSAria II or to FACS-sorting using BD FACSAria III, Bigfoot Spectral Cell Sorter. Cell debris (FSC vs. SSC) and duplicates (FSC-A vs. FSC-H) were removed. To compare high versus low gene-of-interest-expressing cells, unstained cells were used as a negative control. Finally, the 30% of cells with the highest and lowest CD73, ITGA11 or CD151 MFI were sorted for each individual donor and seeded for subsequent differentiation assays or RNA extraction either directly or after 7 days of in vitro expansion.

To investigate SSCs, unstained cells for flow cytometry or fluorescence minus-one (FMO), i.e., staining with all fluorophores except one, for FACS were used as negative controls. Dead cells were excluded using 1 μg/mL DAPI (422801, BioLegend). Due to a high number of utilized fluorophores, compensation was required to correct for fluorescence spillover. SSCs were gated for analysis in Flow Cytometry or FACS using the following strategy of gating: debris and duplicate event removal, CD45^−^, CD235^−^, DAPI^-^, CD31^-^, Tie2^−^, CD146^–^, PDPN^+^, CD164^+^, and CD73^+^. Flow Cytometry quantification was done using FlowLogic version 7.3 or FlowJo version 10.8.1. Cell percentage was determined as the number of positive cells over the total number of singlets, while MFI was defined as the mean value of the positive population.

### Immunohistochemistry and in situ hybridisation

Paraffin-embedded sections were dried at 60 °C for 30 min and deparaffinized through sequential immersion in xylene, 99% ethanol, 0.5% H₂O₂ in ethanol (4 mL H₂O₂ + 236 mL ethanol), 99% ethanol, 96% ethanol, and 70% ethanol (2 × 5 min each). After washing in tap water, antigen retrieval was performed in TE buffer overnight at 60 °C, followed by cooling at room temperature for 20 min. Sections were washed in TBS/Tween (2 × 5 min) and circled with a PAP pen. Blocking was performed with TBS/CAS for 20 min. Primary antibody CD271 (clone MAB367, R&D Systems) was applied at 1:50 dilution (10 µL stock + 490 µL TBS/CAS) for 2 h at room temperature. The negative slide received diluent only. After washing, detection was achieved using polymer anti-mouse HRP (undiluted, DPVM110HRP, BrightVision, ImmunoLogic) for 30 min, followed by TSA Opal 570 (FP1488001KT, Akoya Biosciences) at 1:250 dilution (4 µL stock + 1 mL amplification diluent) for 30 min at 40 °C. Peroxidase blocking was performed with 1% H₂O₂ in TBS (1 mL stock + 32 mL TBS) for 15 min at 40 °C with TBS/Tween wases between steps.

In situ hybridisation (ISH) was performed using the RNAscope Multiplex Fluorescent Reagent Kit v2 (323110, Advanced Cell Diagnostics [ACD]) according to manufacturer’s instructions with minor modifications. Slides were pretreated with custom reagent (300040, ACD) for 20 min at 40 °C, washed in DEPC water, and hybridized overnight at 40 °C with probes targeting CD151 (Hs-1057041-C1, ACD), CD73 (Hs-437931-C2, ACD), and ITGAII (Hs-457751-C3, ACD). Probe mix per slide: 60 µL diluent + 60 µL C1 + 1.2 µL C2 + 1.2 µL C3. Negative control slides received diluent only. Signal amplification was performed through sequential Amp steps (Amp 1–3) at 40 °C, followed by HRP-mediated development and TSA fluorophore deposition: Opal 520 (FP1487001KT, Akoya Biosciences) for CD151, Opal 690 (FP1497001KT, Akoya Biosciences) for CD73, and Opal 620 (FP1495001KT, Akoya Biosciences) for ITGAII, each at 1:250 dilution in amplification diluent for 30 min at 40 °C. HRP blocking was applied between fluorophore steps. Nuclei were counterstained with 1 µg/mL Hoechst (H3569, Invitrogen) for 1 min and slides mounted with ProLong Gold antifade reagent (P36939, Invitrogen) and stored in the dark at 4 °C until imaging.

### Imaging and Data-driven thresholding for signal intensity and cell segmentation

Fluorescent signals were visualized using an Olympus VS200 slide scanner equipped with a Xylis light source (XT720L, X-Cite) and the VS-264C camera at 40× magnification using the extended focal imaging function. Positive and negative slides were examined in Halo and Halo AI (version 3.6.4134.95, Indica Labs). All cells within the marrow were identified using our custom-trained nuclei AI network to ensure accurate cell segmentation. Thresholds for cell classification were established using negative control slides from the combined IHC and ISH. Halo and Halo AI (version 3.6.4134.95, Indica Labs) were used to quantify cells across the marrow and to measure the autofluorescence signal in the negative tissue, without applying any initial thresholds. The resulting intensity data were exported to R, where the 99.99th percentile of each fluorescent channel was calculated and used as the threshold for distinguishing between different cell phenotypes.

### Statistical analyses

All statistical analyzes were carried out in R. Paired samples are connected by lines. *P*-values were calculated using paired two-tailed Student’s *t*-test, linear regression models, unpaired two-tailed Wilcoxon–Mann–Whitney test, hypergeometric test, proportional *z*-test, Fisher’s exact test, or chi-square test. The test used is described in the figure legends. Effect size was calculated using cohen.d function from the effsize package in R. Number of replicates is mentioned in the individual figure legends. Statistics for sequencing-based methods were derived from Seurat,^80^ GOseq,^87^ DESeq2,^84^ or fgsea.^89^

## Data Availability

Raw sequencing data reported in this study have been deposited under the NCBI Gene Expression Omnibus: GSE317531. Processed data and scripts for data processing and visualization to recapitulate the analyses are available at the open science framework: https://osf.io/s8nfb/ and at GitHub: https://github.com/drarauch/InvitroHeterogeneity.
